# Interactions between Street Food and Food Safety Topics in the Scientific Literature—A Bibliometric Analysis with Science Mapping

**DOI:** 10.3390/foods11060789

**Published:** 2022-03-09

**Authors:** Claudio Bellia, Simona Bacarella, Marzia Ingrassia

**Affiliations:** 1 Department of Agricultural Food and Environment (Di3A), Università degli Studi di Catania, 95100 Catania, Italy; c.bellia@unict.it; 2 Department of Agricultural, Food and Forest Sciences (SAAF), Università degli Studi di Palermo, 90128 Palermo, Italy; simona.bacarella@unipa.it

**Keywords:** street food safety, consumer health, sustainable food systems, food security, food consumption

## Abstract

Street food (SF) consists of ready-to-eat food prepared and sold on the street. This food constitutes the food traditions of local populations in many countries of the world. SF characterizes a large number of cities around the world, from New York to Paris, from Palermo to cities of North Africa, China, India and Japan. SF is inexpensive and prepared following traditional methods that meet local consumer preferences, culinary culture and lifestyles. Moreover, SF allows a unique experience for tourists who also want to experience a destination through traditional food consumed on the street together with the locals. Nevertheless, SF is linked to several health hazards. Hence, several studies discussed on the compliance with hygiene and food quality requirements that SF vendors should guarantee, to ensure human health. So far, there is no bibliometric review attempting to provide an objective and comprehensive analysis of the existing scientific documents that simultaneously study the scientific topic of SF linked to that of Food Safety (FS). Therefore, the objective of this paper is to provide a theoretical framework of the interactions between studies on SF and FS topics, in order to discover if the combined topic of “Street Food Safety” (SFS) was investigated as a topic in its own right. A bibliometric analysis was carried out analyzing 276 scientific contributions from the last 21 years, indexed in the Elsevier Scopus database and in the Clarivate Web of Science database. The results showed a very strong interaction between the two topics and many others in several scientific sectors; In particular, the topic of SFS involves many disciplines of social sciences. The results highlight that the scientific topic of SFS exists but not consciously, and it is believed that the research interest in this topic can grow considerably in the coming years, also because of the current COVID-19 pandemic situation that we are experiencing.

## 1. Introduction

Street food (SF) belongs to the culture of many populations around the world. In particular, it is a food usually eaten in many cities of continents such as Africa, India, Asia and Latin America [1]. Street food and street food vendors are particularly popular in under-developed regions and countries, where they constitute a relevant part of their economy [2,3,4]. However, SF represents a fundamental part of the eating habits in many cities of the most developed countries. As a fundamental part of the local culture, SF is often an attraction for visitors/tourists in many cities and countries of the world (e.g., New York in the USA, Paris, Vienna, Lisbon, Madrid, Rome and Palermo in Europe, but also in Greece, Turkey and other countries of the Mediterranean Basin) [5,6]. According to the FAO definition, street foods are ready-to-eat foods and beverages prepared and/or sold by vendors or hawkers especially in the streets and other similar places. SF includes a very wide quantity of products of animal and vegetable origin, grilled or fried, seasoned and served according to local traditions [7].

SF is gaining increased popularity in both under-developed and developed countries [8]. It is clear that the consumption of SF is due to different factors depending on whether we are referring to a more developed or non-developed country. In fact, for millions of low- and middle-income consumers in urban areas of developing countries, street food is a substantial component of their daily diet. For them, street foods may be the cheapest and most accessible way to have a nutritionally balanced meal outside of the home, provided that the consumer is knowledgeable and capable of selecting the right combination of foods. Moreover, in developing countries with inadequate education or skills, preparing and selling street food is a reliable source of income. Differently, in some developed countries, SF is considered an additional tourist attraction. Think about the so-called “food & wine” tourism that, for example in Italy, attracts millions of interested tourists every year to try the typical street food and live a complete and inclusive experience together with the inhabitants of the place they are visiting [9,10]. SF is a conscious way to show tourists and visitors part of the cultural identity of the local population, the so called “food identity” [11]. SF allows a unique experience for tourists who also want to experience a destination through traditional food consumed on the street together with the locals [12]. Street food vendors, along the road, become a meeting point between locals and visitors, between tasting and storytelling [9]. This point was addressed by some authors that described street food as a “diversified touristic attraction and offer” [9], as it allows, at the same time, tourists to explore culinary traditions of the visited country/city and the territory of origin of the products tasted. This contributes to have a unique experience of travel and knowledge of places and cultures of which the food itself contributes to maintaining a positive memory [13]. It is linked to broader concept of experience that include cultural heritage, food quality, local food products, health, and food safety [12].

However, street food can also exert negative impacts in the long term on human health and the environment, such as pollution, damage to biodiversity, etc. [14]. Additionally, SF has some problems connected to food safety, hygiene, and food quality, which can compromise the health of consumers, both during the immediate consumption and in the long term (due to cooking and preparation methods) [15]. According to FAO, local governments, international organizations, and consumer groups are becoming aware of the socioeconomic relevance of street foods, as well as the risks that come with them [15]. Food safety is the primary concern, but other issues such as sanitation (waste accumulation in the streets and clogged wastewater drains), traffic congestion in the city, including for pedestrians (occupation of sidewalks by street vendors and traffic accidents), illegal occupation of public or private space, and social issues have also been raised (child labor, unfair competition to formal trade, etc.). In many parts of the world, the possibility of serious food poisoning outbreaks linked to street foods remains a concern. A lack of understanding of the causes of food-borne illness among street food vendors is a problem. The relationship between street food (SF) and food safety (FS) is particularly interesting, due to its importance the global economy. In recent decades, the subject of FS has taken on a significance linked to the economic development of the countries to which it refers [16,17]. In fact, FS is generally associated with the probability of contracting diseases due to the qualitative characteristics of the food or to the lack of compliance of SF vendors with the minimum health and hygiene requirements [18]. For example, it is important for consumers to adopt some caution, such as avoiding raw foods, because the preservation methods required to keep them fresh are rarely possible on the street, or avoiding drinking running water [14,15]. It is also important that the surfaces on which food is processed and cooked are well cleaned and that fresh foods are stored in refrigerators. Finally, the handling of money with hands before serving or eating the food is another problem [3,7,14].

Due to the COVID-19 pandemic and the sanitary crisis, the consumers’ behavior in regard to food consumption and purchasing has significantly changed, particularly in urban cities in the most developed countries in the world [19]. Because of the persistence of lockdowns and the fear of the spread of contagions, consumers avoided traditional neighborhood markets of fresh foods, for the need of social distancing, and reduced the consumption of SF, preferring to buy food at supermarkets or grocery stores, often online, or requesting for home delivery [20]. In addition, it was observed that consumers stocked up essential items at home, and these behaviors largely remained even after the reopening [21]. Additionally, the SF economic sector was significantly affected, not only with regard to the lower number of local consumers but also for the absence of tourists/visitors, which is also an economic loss for SF sellers [22,23].

Because of the above-explained variation of food safety with geography, the term “Street Food Safety” calls for stronger contextualization and substantiation.

Despite the importance of studying issues related to the healthiness and safety of SF, notwithstanding scientific progress made in this field, bibliometric analysis of scientific works published on these topics is still lacking. Literature on SF begins around the end of nineties [24], and some studies have been conducted on SF and FS about specific topics such as hygienic practices [25,26], food allergens [27] and others, such as the tourism economic perspective for small enterprises [28,29,30]. Nevertheless, there is no bibliometric analysis attempting to provide an objective and comprehensive analysis of the existing interactions between these two topics. Abrahale et al., which presents a scoping review concerning SF and its socio-health aspects [31], and another Bouafou et al., [32] on food science and technology, and there are a few recent works in the literature on SF around the world and the risk perception toward FS in SF [33,34].

Therefore, the study of the links between the scientific topics SF and FS appears of interest for researchers that want to know how different disciplines, fields, expertise and documents/authors relate to each other while investigating those two topics, and discover any existence of a further scientific topic related to them, that is, “Street Food Safety” (SFS). This study focused on the two scientific topics of research, SF and FS, with the aim to highlight any strand of research concerned with studying the combined topic SFS or the two topics separately. Moreover, this study aims to identify which scientific fields emerge in which these two topics (SF and FS) are studied together, or in which field the specific SFS topic is studied. Specifically, the object of this paper was to discover, based on the empirical evidence, any existing objectively demonstrable interaction between the scientific topics of SF and FS, and the existence of the scientific field of research regarding “Street Food Safety”. The research questions were:

RQ1. What relation is there between street food and food safety topics in the scientific literature?

RQ2. What is the current scientific literature analyzing the “derived” composed topic “Street Food Safety”, by focusing on leading scientific contributions?

To answer these questions, we conducted a bibliometric analysis of the literature based on scientific mapping [35,36] in order to highlight any aspect of the bibliometric links between SF topic and FS topic and any existence of the topic “Street Food Safety” highlighted by authors who have studied this theme in any scientific sector (not necessarily the field of exact sciences). Results provided an interesting insight into current research trends and potential directions for future research.

## 2. Materials and Methods

Following the objectives of this study, it was considered more appropriate to combine two methodological approaches. First, the PRISMA protocol [35], a proven procedure in the field of “systematic literature reviews” and “meta-analyses” that provides transparency and replicability to the review. By the use of this procedure, it was possible to obtain a representative set of scientific documents regarding the topics of “Street Food”, “Food Safety” and “Street Food Safety”. Moreover, using scientific mapping analysis (or bibliometric mapping analysis, using “keywords” analysis, co-citation analysis and “co-occurrence analysis”, it was possible to highlight how disciplines, fields, expertise and individual documents or authors relate to each other [34,37]. Both techniques have been used in previous works [35,36]. Keyword and co-occurrence investigation provided a theoretical framework of the relations between studies on SF and FS topics, and the combined topic of “Street Food Safety” (SFS), particularly the interactions between the three topics in diverse disciplines and the most interesting studies published related to these research themes (co-citation analysis).

### 2.1. Bibliometric Analysis

An extensive literature search, a chronological/conceptual review of international literature, was conducted in March 2021 and in February 2022, with the aim to find out scientific documents published from 2000 to 2021 (included) discussing the relations between the topics “Street Food” and “Food Safety” [37,38,39,40]. This analysis was carried out on documents published from 2000 because, during the last 21 years, consumers and the scientific community have started to take more into consideration the problems linked to the risks to human health of the consumption of certain foods. In addition to that, the importance of the controls on foods quality and food safety have increased since that time, especially in developing countries, where the issue of food safety is still a very important public health issue.

The Clarivate Web of Science and the Elsevier Scopus databases were used for this study. The choice to combine these two databases allowed to reduce greatly any type of bias in the selection of data sources, as it ensured an adequate number and model of document classification according to the purpose of this study. In fact, it was thought to have a sufficient number of medium to high quality documents covering the natural and social sciences, both sectoral and interdisciplinary, within a formal or applied sciences system [41]. Moreover, the choice of the two databases appeared the most suitable to have a more heterogeneous significant sample, because of the high number of journals of medium–high quality indexed. Among all the scientific database, Google Scholar was excluded because it indexes many non-scientific papers. Finally, the PubMed database specializes in “biomedical literature”, and was therefore too sectorial, and not suitable for our investigation. Regarding the language used, there were selected scientific documents published in all languages.

The first step of the used method concerned the research and selection of the studies under investigation in the two databases.

**Figure 1:**
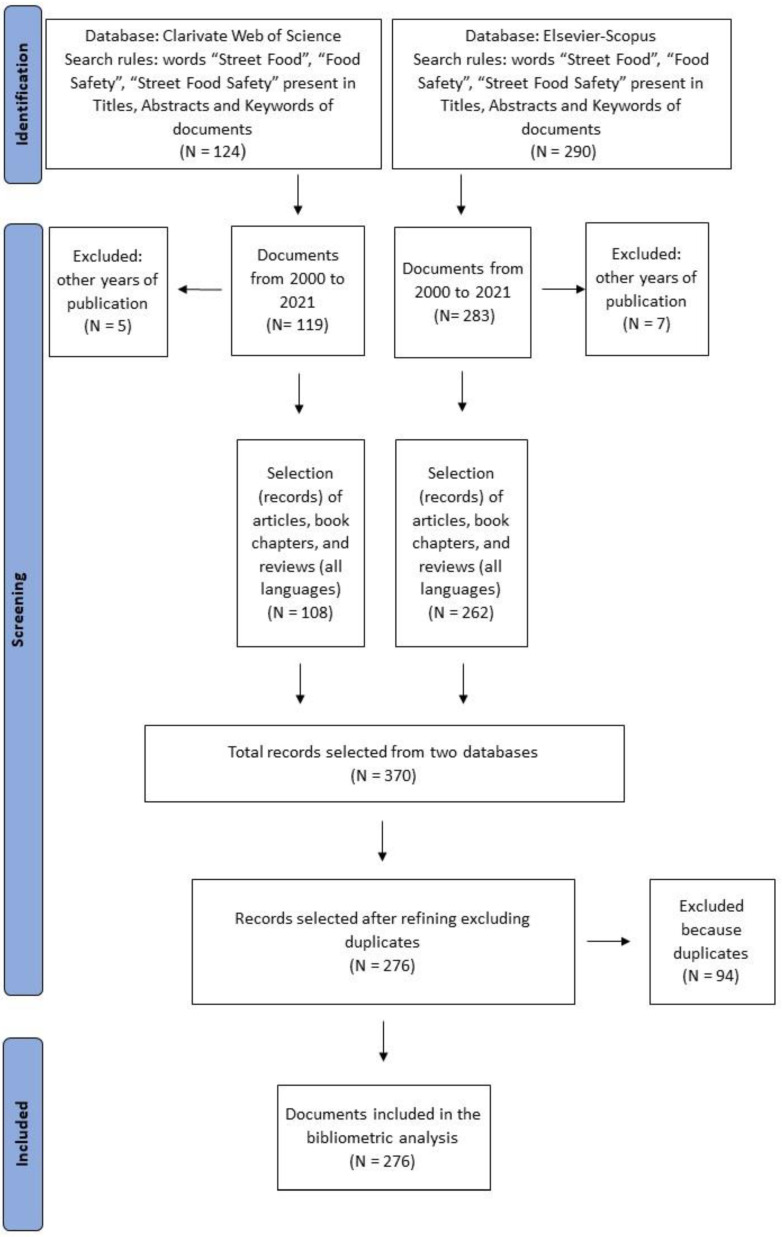
Inclusion and exclusion criteria using PRISMA protocol. Final number of documents observed N = 276.

Usually, PRISMA protocol is used in systematic literature reviews to show a series of successive stages that allowed us to select the relevant documents for the study. In this study, the PRISMA protocol was applied (Figure 1) because, similarly to other bibliometric reviews [34], it appeared suitable to simplify the understanding of the study design followed, providing transparency and replicability of the review.

The initial search was screened and refined using the Elsevier “Search within” function, applying multiple combinations with article titles, abstract and keywords. The search terms were selected in line with previous research in the field of street food [35,36] used Boolean strategies that are: “Street Food” AND/OR “Food Safety” OR “Street Food Safety”. Through this procedure, a first database was created (identification).

After the first step of identifying the sample for analysis, the times when pairs of articles within the sample were cited in the other scientific documents within the entire Elsevier Scopus database were counted [35,36].

During the last twenty-one years, there has been a strong increased interest from academics for the topics concerning street food and/or food safety; therefore, it was decided to consider for this study all documents published from 2000. The extraction rules were limited the extrapolation only to scientific articles, books/book chapters and reviews fully published in Journals between 2000 and 2021 (included), written in all languages. It is possible that after 2020, due to the COVID-19 pandemic, the interest of researchers on street food safety increased further; therefore, this study can be continued into the future.

Duplicates and documents not providing original data (e.g., opinion papers), and study protocols were excluded. So, the initial database consisted of N = 414 documents.

From the initial database of documents, it was created a second database (N = 370), after having checked the consistence of contents with the words searched as representative of the research topics (SF, FS and SFS).

The final database consisted of N = 276 documents eligible for the bibliometric analysis, which included 231 articles (83.69%), 30 book chapters (10.86%) and 15 reviews (5.43%).

A first analysis of the selected documents was performed using basic quantitative statistics in order to know the characteristics of documents included in the final database (FD), such as year of publication, authors, type of document, document citations, scientific areas, countries, etc.

Subsequently, with the frequencies obtained some multivariate statistical techniques were applied, with the aim to identify the existent structure of connection among the documents based on the terms considered representative of the observed topics (SF, FS and SFS). For this BA, the software Microsoft VOSviewer (version 1.6.16.) was used. This (Van-Eck & Waltman, 2011, 2020) software allows researchers to create two-dimensional maps for the construction and visualization of bibliometric networks [42,43]. It enables us to build keyword networks extracted from scientific literature, using a text mining functionality. Using this software, it was possible to carry out the scientific mapping.

### 2.2. Science Map Analysis

The so-called scientific mapping, or “science maps”, are spatial representations that help to visualize the relations that arise among documents, based on links between authors, bibliographic references, journals, disciplines, and specific words. This type of network analysis allows researchers to search into the content of documents through the co-occurrence of “specific words” (SW) (or key-words). This methodology is especially suitable to know the intellectual structure of a specific research field or topic of interest. According to previous studies [34,36], the SW used in the content of documents and those contained in titles, abstracts and keywords are essential for the identification of significant topics within a specific research topic of issue.

The visual representation of the Science Map can be obtained using any bibliometric software [34,36]. For this study, the VOSviewer [42,43] appeared the most suitable because it provides a high-quality overview of targeted research topics. However, the information underlying the visual representations provided by the software is certainly of interest. In this study, due to the graphical power of the software, and the meaning of each of the visual representations, we wanted to know the relationships between the topics of “Street Food” and “Food Safety” and any scientific interest in the topic identified as “Street food Safety”, despite the possible non-use of the specific keyword.

Thanks to VOSviewer, the importance of a SW (node) is represented by its relative position in the network. The software calculates the importance of all the SW selected and displays the largest possible number of thematic networks. The node size represents the importance of those words, based on the frequency of occurrences [43]. Links between nodes denote the number of times words appear together, and the thickness of the link means the strength of the link.

Special words’ map analysis and links’ strength analysis (co-occurrence analysis) were performed. The co-occurrence analysis allowed us to discover any relations arising among the documents selected and the entity/strength of these relations (links), which consists of distances and label size, based on the frequency of words co-occurrences. Therefore, VOSviewer provided a visual representation of the networks among the three topics investigated: street food, food safety and street food safety.

## 3. Results

This study provides the first bibliometric analysis (BA) and classification framework of the existing literature about street food and food safety and the bibliometric links between these two scientific topics. The aim was to know if the topic “Street Food Safety” was an existing cross-sectorial topic of interest for researchers, and what were the main scientific areas in which this issue was studied as relevant topic.

The final database (FD) consisted of 276 documents published and indexed in the two databases in the period between January 2000 and December 2021.

### 3.1. Analysis of the Selected Manuscripts: Data Description and Classification

Through this BA, it was possible to classify the documents observed by author names, title, year of publication, document’s type, journal’s title, and number of citations by other scientific documents. Despite the fact that there are several publications documenting these topics in various parts of the world, the most frequently cited publications are, primarily, cases studied in Africa and South America. Most of them are documents focusing on specific case studies on food safety of street food, with some theoretical contribution to existing theories. This might be because the problem of food safety of street food is very relevant in those world areas.

**Figure 2:**
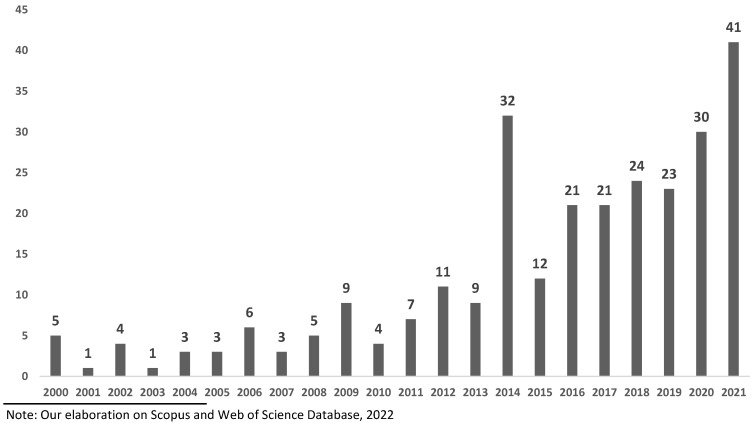
Evolution of the number of articles (276 documents observed) published per year.

Results shows a progressive increase, albeit non-linear, from 2000 until 2021, of publications regarding topics of street food and food safety, as shown in Figure 2.

It can be noticed that the number of scientific documents published increased significantly in 2014 (11.59% of the total 276 documents observed). Specifically, most of the published documents on the studied topics (204 documents, equal to 73.91% of total documents observed) were published between 2014 and 2022, with the exception of 2015, where there were only 12 articles, but that year was the only one not in line with the trend of the last 8 years.

**Figure 3:**
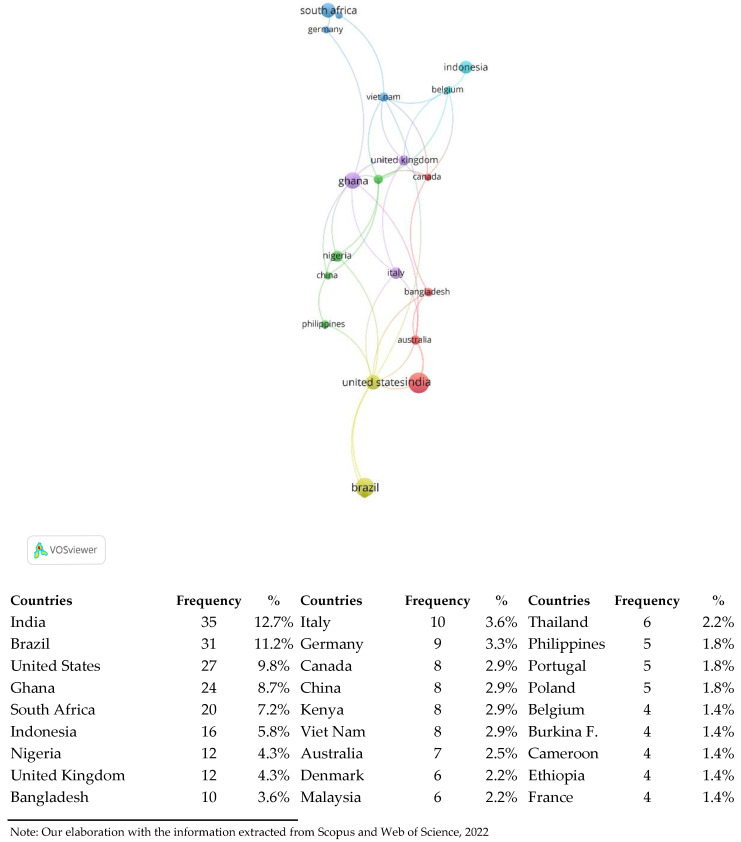
Frequency of the scientific papers about street food and food safety by focused country.

Figure 3 shows the number of scientific documents on the topics SF and FS published from 2000 to 2021, by focused country.

It can be seen that India, with 35 documents on SF and/or FS (12.68% of the 276 documents), was the country with the highest number of interested authors; following India were Brazil, USA and Ghana, with more than 20 documents, South Africa with 20 documents (7.2% of total 276 documents), and then Indonesia (16 documents, 5.8%), Nigeria (4.3%), UK (4.3%) and Bangladesh (3.6%). This result highlights that generally, in the occidental countries and specifically in the EU, the topic of food safety for street food is not considered an issue, possibly thanks to specific strict regulations to preserve food quality and to maintain a high level of sanitary standards. Moreover, street food is much more frequently sold and consumed as a regular meal in developing countries than in those more developed [25,29,44].

**Figure 4:**
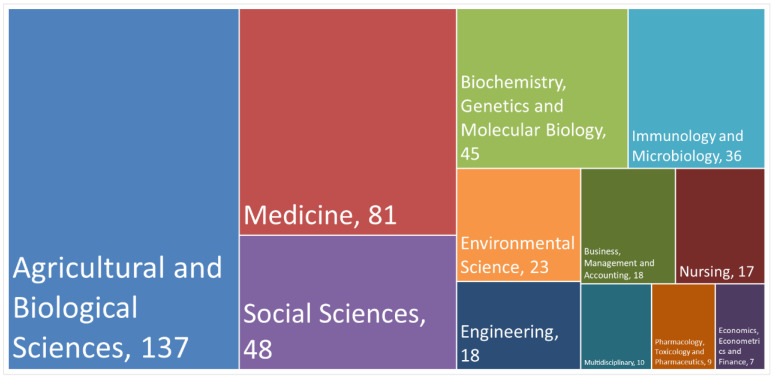
Number of published documents by research field.

Figure 4 shows a high heterogeneity of documents and the diversity of the research fields where the topics SF and/or FS were studied (12 research field in total), according to the number of documents published in the selected databases.

The five research fields with more documents were: agricultural and biological sciences (included agricultural economics) with 137 documents—49.63% of the total (276); medicine (81 documents) 29.34% of the total; social sciences (48 document on 276) 17.39% of the total database; biochemistry, genetics and molecular biology 16.3% (45 documents) and immunology and microbiology 36 documents—13%. Following these were environmental science, engineering, business management and accounting, nursing, multidisciplinary, pharmacology and economics, econometrics and finance. In this scenario, it is interesting to observe that social sciences is a field where SF and FS were studied relatively frequently (almost 20% of the total documents observed), and also in combination.

Table 1 shows the top 20 most cited documents in the SD. Specifically, the manuscript of Mensah et al. (2002) [44] has the highest number of citations, 202 citations, among all the publications observed in the FD on an article analyzing safety and healthiness of street food in a city of Ghana. It is followed by a review of Rane (2011) [45], with a citation frequency of 103, that analyzes the problems of risks related to street food. By observing all the articles in this Table, it is possible to notice that the most recurring themes are the relationship between street food and the hygiene of the place where it is sold and consumed, and the fact that it has become an important public health issue in the developing continents. Other aspects investigated by the authors are related to the socio-economic role of the street food vendors. Most of these are documents published on very sectorial journals concerning food safety, knowledge and risk, etc. such as the journal “Food control”, which deals with these issues.

### 3.2. VOSviewer Results: Science Maps

**Figure 5:**
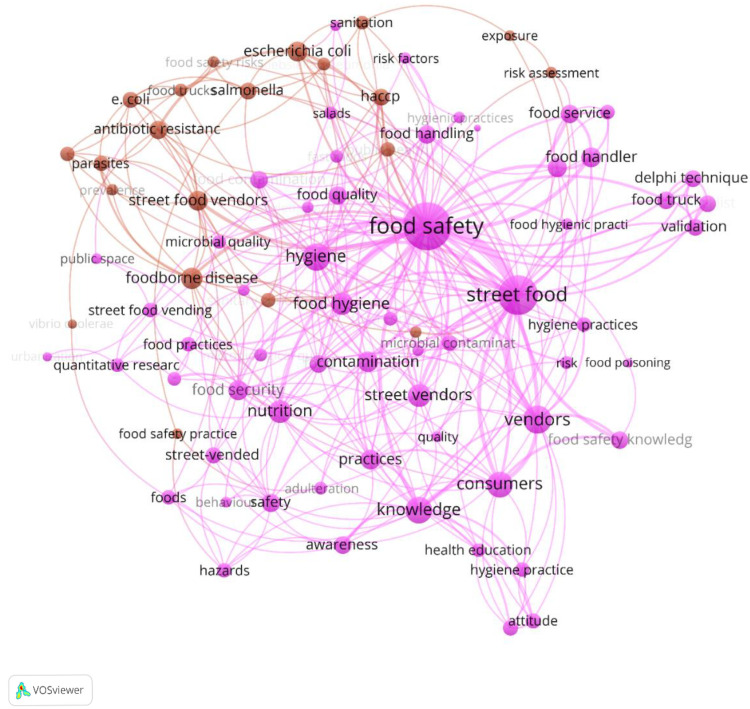
VOSviewer keywords analysis and co-occurrences in the selected documents.

All the SW contained in the titles, abstracts and keywords of the 276 selected documents were selected, and they consisted of 797 words to be analyzed (census) [42,43]. Previously, the database was manually cleaned of duplicates, plural words, and other words considered not relevant for this study (e.g., “analysis”, “theory”, “study”, “review”, etc.). At the end, the words database consisted of 110 words. The 110 selected words were analyzed using the VOSviewer software, with the final result a science map showing the relationships between the various SWs, and among them, plus their association through the thematic clusters highlighted (Figure 5).

In the map, it was possible to see clearly the bibliometric links between the two topics observed (SF and FS) and all the other correlated SW in the selected documents. As shown in Figure 5, following the “VOSviewer keywords analysis” each node is a word, the larger the node, the higher the frequency of occurrence of the word identifying it. The lines between two nodes (links) and their thickness indicates the co-occurrence of two words in the same document. Specifically, links between/among nodes indicates that two or more words appear in the same document, and the thicker the line, the higher the frequency of word co-occurrence [42,43]. Moreover, the thickness of the link indicates the strength of the link based on co-occurrences. The network connections show the words that appear together more frequently in the analyzed documents. Thus, it is possible to identify clearly the most prevalent/important research topics discussed in the research/study documents, according to their authors.

The “VOSviewer keywords analysis” [42,43] highlighted two big clusters based on the 110 words analyzed. These words are displayed in the science map with 306 links that connect them to each other, highlighting the multidisciplinary approach to issues related to SF and FS.

The nodes’ size indicates the weight of the SW, i.e., its occurrences. Food safety is the biggest node, followed by Street Food. The word “Food Safety” appears in almost half of the documents in the selected database; consequently, this node is the biggest one, having the highest number of co-occurrences with the other linked words, and it clusters the largest number of correlated topics.

The second biggest cluster is that of the topic “Street Food”, which is obvious considering that these two words were the ones selected as “key-selection words” in the first phase of sampling in the Elsevier Scopus database. However, the interesting thing is the strength of the link between these two topics, which shows that there is high interest of research for the two combined topics of SF and FS. Furthermore, the nodes and the links discovered show what are the other principal topics studied in combination with SF and FS, this highlights the scientific sectors and issues wherein these two topics are studied together.

Very few documents (1.5% of the total documents observed) contained the word “Street Food Safety” in their titles, abstracts and keywords, regarding microbial safety, social determinants of health, public health intervention and food safety education in elementary school students. Due to the low frequency with which this word has been detected, it does not appear in the visual representation provided by the software.

It can be observed that the strongest connections of FS are with topics related to human health issues, such as “food”, “hygiene”, “nutrition”, “hazard” and “contamination”. Moreover, the most important connections with SF are other topics related to consumers and their awareness of SF hazards and SF characteristics, e.g., topics such as: “consumers”, “knowledge”, “street vendors”, “hazard”, “hygiene practices”, “women” and “nutrition”.

Table 2 shows the most relevant words found in the analysed documents based on occurrences and links’ strength.

From the total of 276 selected articles and review documents, the keyword “Food Safety” was found in 79 documents, reflecting a percentage of 28.6% of the total number of documents, with the highest strength of the links (LS 180) that correlate it with other topics. It is not surprising that this topic is followed by “Street Food” with a frequency of 48, which is 17.2% of the 279 selected articles and a link strength (LS) of 104. Other frequent SW are “hygiene” (8.3%) and “vendors” (4.3%). “Consumers”, “Food hygiene” and “Es. Coli” (2.9%) are moderately frequent, although not as relevant as the first ones. These results show that the other SW do not have their own capability to be a multidisciplinary topic, because their combination with any of the other word observed is modest. On the other hand, these findings show the relevance of combination among SF and FS topics and other topics such as “consumers”, “food hygiene”, “knowledge”, “nutrition and diseases” and the way in which the issue of food safety of SF is studied.

**Figure 6:**
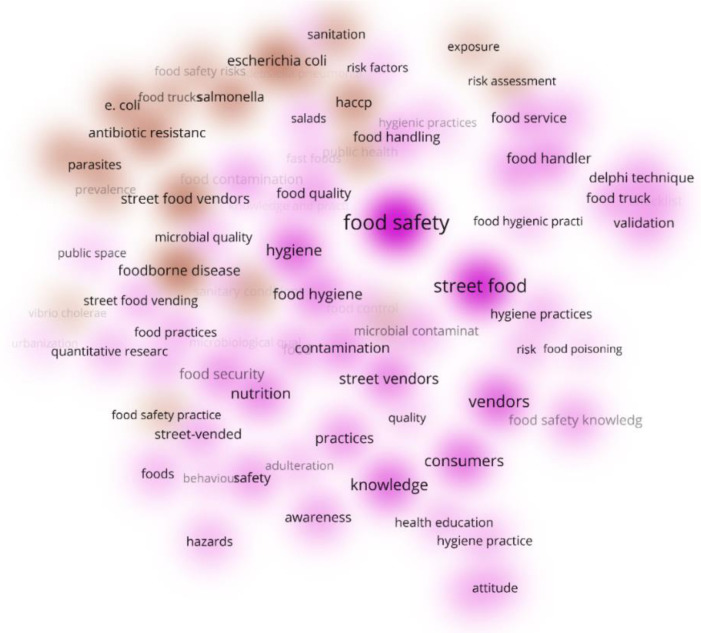
Density visualization performed by the VOSviewer program.

Figure 6 displays the clusters’ density. This Figure shows the same bibliographic findings of Figure 5, but without networks. In this Figure, the intensity/density of the color reveals the weight of each cluster measured by the number of items belonging to that cluster in the neighborhood of the point [36,37].

The density of Cluster 1, with 58 words (data not shown) (63%), addresses “Food Safety” issues. Cluster 2 (density of 33 words) addresses street food issues, with a weaker cooperation strength than that of Cluster 1. The similar colors and their intensity show cooperation strength of the words, which identifies the topics studied in the same document.

Nevertheless, the interesting finding that emerges from this result is that the highest density is located in the proximity of the word FS and SF. Therefore, it is possible to demonstrate that a line of research, or a field, that studies issues and themes regarding “*Street Food Safety*” exists, despite the fact that this “key”-word is used very rarely in the selected documents.

## 4. Discussion

Through this pilot research, using a bibliometric analysis of scientific documents on the topics “Street Food” and “Food Safety”, it was attempted to discover the scientific interactions and connections between the two topics.

The first finding that emerges from the results is that the interest to pursue research related to food safety within street food sector has significantly increased during the last 21 years; in fact, between 2000 and 2021, the number of articles on street food, food safety and their connection increased progressively (Figure 2). The aim of the studies observed was to study how to increase food safety and hygiene during preparation and consumption of street food and also the consumers’/vendors’ knowledge about this issue [64,65,66,67,68,69]. Although the SFS covers a wide range of subject categories, a large proportion of the publications were related to street food and food safety separately. Specifically, it is possible to note that in the Cluster 1, according to the topics found [51,70,71], groups all the documents where the issue of food safety is discussed, and Cluster 2 groups documents about street food. Interactions between the street food topic and food safety, and other relevant research topics, show that relations are mainly related to economic topics [72,73,74,75].

The analysis of the 279 selected documents highlights the increasing interest of researchers for issues of SF for human health, and thus the importance of FS for global and local policies [2,74,75]. Almost all public health challenges are discussed with regard to specific geographic areas, so there is an increase in the number of journals in these fields, journals such as *Foods*, *Food Control*, *International Journal of Food Microbiology*, etc.

The interactions between SF and FS can be also found by observing the other research sectors linked to them, such as “food allergies”, “diseases and hygiene”, “nutrition” and “food contamination” issues [1,55,76,77]. Topics related to nutrition, hygiene practices, food safety knowledge, hazard, contamination, microbiological quality, risk assessment, and others were the most discussed in the identified articles, showing some common issues of great concern. Much research has been conducted in developing countries [2,76,77].

Moreover, street food and food safety issues are closely related to the food system topics. In fact, most of the documents were included in the research area “Agricultural and Biological science” in which there are journals dealing with issues of “Agricultural Economics”. SFS research involves about 84% of all subject categories, meaning that there are numerous perspectives of research and scientific studies on the specific topic of SFS. The study found that SFS topics are studied mainly with regard to developing countries, such as India, Brazil and Ghana, which have undeveloped economies and little investment in scientific research. Nevertheless, this topic is also covered with regard to the USA. In fact, SFS cannot disregard based on political, socio-economic and environmental regional characteristics; nevertheless, the current bias in geographical distribution of SFS research seems acceptable and thus it allows the generalization of findings.

It is believed that further research targeting developed countries is needed [66,78,79,80,81], and also the analysis of documents written in different languages other than English and indexed in other scientific databases, because they address these themes and topics from different point of view and with a more systemic approach.

This pilot study, with BA [81], contributes to the actual research showing a new latent field of research on “Street Food Safety”, because of the revealed closed links between the two topics, particularly in the research areas of medicine, agricultural economics, biological sciences, and social sciences. Therefore, this opens up a new potential research field in the literature. This novelty of findings may be of interest for other authors interested in studying the Street Food Safety issues from a broader interdisciplinary perspective, and not necessarily linked only to food hygiene or public health issues. In fact, results clearly show that there is an existing research interest on the SFS topic that includes other topics of cross-sectoral interest, studied primarily by academics of social sciences (agricultural economics, agricultural politics, economics, etc.), e.g., food quality topics, quality certifications, of the traceability and retraceability of a food product, block-chain, etc.

### Limitations and Future Lines of Research

The aim of this study was to contribute to the knowledge of the specific broad interest research sector of *Street Food Safety*. This pilot study, using BA [80,81], opens the door for future analysis by combining other databases of research documents that can confirm or contrast our results. However, the results of this study have some limitations that need to be addressed in future studies, and do not offer a unique view of reality. In particular, the most important is that, although we were able to obtain objective results on the topic of SF and FS and with regard to the discovered latent topic of Street Food Safety, some reasons behind these results are still not explained. Therefore, a more detailed analysis of the studies about the topic of Street Food Safety in developed countries appears useful in adjusting the geographic distribution of research documents analyzed. Therefore, to address this limitation, which is typical of BA [80,81], future research could be carried out with the using statistical methods suitable for explorative studies in social sciences. Additionally, these results could be integrated with a systematic literature review. However, the complexity of the phenomenon and its importance demonstrates that more research need to be conducted on the extensive topic of Street Food Safety.

## 5. Conclusions

This study is the first bibliometric analysis and classification framework to review systematically the status of the existing literature on the topic of “Street Food Safety” highlighting the bibliometric links between street food topic and food safety topic. The aim was to know if the topic “Street Food Safety” was an existing cross sectorial topic of interest for researchers, and what were the main scientific areas in which this issue was studied as relevant topic. A quantification of the increase in SFS-related interdisciplinary topics was made in order to highlight the importance that the scientific topic of SFS in acquiring progressively during the last 21 years (from 2000 to 2021).

Through the co-occurrence analysis of research countries and journals, and to citation analysis [35,36,81] we found that most research is performed in developing countries and that these countries have similar problems related to food safety of street food and health of regular consumers of street food. Furthermore, keywords analysis, co-occurrence analysis and cluster analysis, revealed the current research focus and trends [36]. Researchers are focused on one main aspect: food security related to street food preparation and consumption in the daily diet of consumers. This finding highlights that there is a developing field of research on the topic “Street Food Safety” and on all the other topics related to it. Specifically, the research sectors where authors investigate on this topic are those of the social sciences where topics are studied using an integrated and systemic view. Therefore, more research should be carried out in the future investigating documents that focus on developed countries also using other database to complete the analysis.

## Figures and Tables

**Table 1:** Rank of first 20 most cited articles.

Rank	Authors	Title	Year	Type	Journal	Cited by
1	Mensah P., Yeboah-Manu D., Owusu-Darko K., Ablordey A. [44]	Street foods in Accra, Ghana: How safe are they?	2002	Article	Bulletin of the World Health Organization	202
2	Rane S. [45]	Street Vended Food in Developing World: Hazard Analyses	2011	Review	Indian Journal of Microbiology	103
3	Zanin LM., da Cunha DT., de Rosso VV., Capriles VD., Stedefeldt E. [46]	Knowledge, attitudes and practices of food handlers in food safety: An integrative review	2017	Review	Food Research International	89
4	Omemu A.M., Aderoju S.T. [47]	Food safety knowledge and practices of street food vendors in the city of Abeokuta, Nigeria	2008	Article	Food Control	88
5	da Cunha D.T., Stedefeldt E., de Rosso V.V. [48]	The role of theoretical food safety training on Brazilian food handlers’ knowledge, attitude and practice	2014	Article	Food Control	81
6	Rheinländer T., Olsen M., Bakang J.A., Takyi H., Konradsen F., Samuelsen H. [49]	Keeping up appearances: Perceptions of street food safety in urban Kumasi, Ghana	2008	Article	Journal of Urban Health	76
7	Mosupye F.M., Von Holy A. [50]	Microbiological hazard identification and exposure assessment of street food vending in Johannesburg, South Africa	2000	Article	International Journal of Food Microbiology	73
8	Nyenje M.E., Odjadjare C.E., Tanih N.F., Green E., Ndip R.N. [51]	Foodborne pathogens recovered from ready-to-eat foods from roadside cafeterias and retail outlets in alice, eastern cape province, South Africa: Public health implications	2012	Article	International Journal of Environmental Research and Public Health	59
9	Muyanja, C., Nayiga, L., Brenda, N., Nasinyama, G. [52]	Practices, knowledge and risk factors of street food vendors in Uganda	2011	Article	Food Control	59
10	Barro, N., Bello, A.R., Savadogo, A., Ouattara, C.A.T., & Iiboudo, A.J. [53]	Hygienic status assessment of dish washing waters, utensils, hands and pieces of money from street food processing sites in Ouagadougou (Burkina Faso)	2006	Article	African Journal of Biotechnology	59
11	Hanashiro A., Morita M., Matté G.R., Matté M.H., Torres E.A.F.S. [54]	Microbiological quality of selected street foods from a restricted area of São Paulo City, Brazil	2005	Article	Food Control	58
12	Samapundo, S., Climat, R., Xhaferi, R., Devlieghere, F. [55]	Food safety knowledge, attitudes and practices of street food vendors and consumers in Port-au-Prince, Haiti	2015	Article	Food Control	57
13	Idowu, O.A., Rowland, S.A. [56]	Oral fecal parasites and personal hygiene of food handlers in Abeokuta, Nigeria	2006	Article	African Health Sciences	49
14	Cortese, R.D.M., Veiros, M.B., Feldman, C., Cavalli, S.B. [57]	Food safety and hygiene practices of vendors during the chain of street food production in Florianopolis, Brazil: A cross-sectional study	2016	Article	Food Control	46
15	Lues, J.F.R., Rasephei, M.R., Venter, P., Theron, M.M. [58]	Assessing food safety and associated food handling practices in street food vending	2006	Article	International Journal of Environmental Health Research	46
16	Liu, Z., Zhang, G., Zhang, X. [59]	Urban street foods in Shijiazhuang city, China: Current status, safety practices and risk mitigating strategies	2014	Article	Food Control	41
17	von Holy, A., Makhoane, F.M. [60]	Improving street food vending in South Africa: Achievements and lessons learned	2006	Review	International Journal of Food Microbiology	41
18	Samapundo, S., Cam Thanh, T.N., Xhaferi, R., Devlieghere, F. [61]	Food safety knowledge, attitudes and practices of street food vendors and consumers in Ho Chi Minh city, Vietnam	2016	Article	Food Control	40
19	Choudhury, M., Mahanta, L., Goswami, J., Mazumder, M., Pegoo, B. [62]	Socio-economic profile and food safety knowledge and practice of street food vendors in the city of Guwahati, Assam, India	2011	Article	Food Control	40
20	Barro, N., Bello, A.R., Itsiembou, Y., (…), De Souza, C., Traoré, A.S. [63]	Street-vended foods improvement: Contamination mechanisms and application of food safety objective strategy: Critical review	2007	Review	Pakistan Journal of Nutrition	39

Note: Our elaboration on data from Elsevier Scopus and Web of Science Database, 2022.

**Table 2:** Rank of the 15 most frequent keywords, occurrences and links’ strength.

Keyword	Occurrences	Total Links’ Strength (LS)
food safety	79	180
street food	48	104
hygiene	23	56
vendors	12	41
consumers	8	33
food hygiene	8	26
eschericchia coli	8	18
knowledge	7	35
street food vendors	7	16
street vendors	6	22
food quality	6	13
nutrition	5	24
food handler	5	16
antibiotic resistance	5	14
safety	5	14

Note: Our elaboration of data extracted from selected documents.

## Data Availability

Not applicable.

## Abbreviations

The following abbreviations are used in this manuscript:

SF	Street Food
FS	Food Safety
SFS	Street Food Safety
BA	Bibliometric Analysis
